# The Characteristics and Functionalities of Mobile Apps Aimed at Patients Diagnosed With Immune-Mediated Inflammatory Diseases: Systematic App Search

**DOI:** 10.2196/31016

**Published:** 2022-03-04

**Authors:** Rosa Romero-Jimenez, Vicente Escudero-Vilaplana, Esther Chamorro-De-Vega, Arantza Ais-Larisgoitia, Maria Elena Lobato Matilla, Ana Herranz-Alonso, Maria Sanjurjo

**Affiliations:** 1 Instituto de Investigación Sanitaria Gregorio Marañón Hospital General Universitario Gregorio Marañón Madrid Spain

**Keywords:** immune-mediated inflammatory disease, mobile app, mHealth, mobile health, chronic disease, disease management, outcomes, functionality, quality, patient education, health outcomes, reliability

## Abstract

**Background:**

Immune-mediated inflammatory diseases (IMIDs) are systemic conditions associated with a high social and health impact. New treatments have changed the prognosis of IMIDs and have increased patient autonomy in disease management. Mobile apps have enormous potential to improve health outcomes in patients with IMIDs. Although a large number of IMID apps are available, the app market is not regulated, and functionality and reliability remain uncertain.

**Objective:**

Our aims are to review available apps for patients with IMIDs or caregivers and to describe the main characteristics and functionalities of these apps.

**Methods:**

We performed an observational, cross-sectional, descriptive study of all apps for patients with IMIDs. Between April 5 and 14, 2021, we conducted a search of the App Store (iOS) and Play Store (Android) platforms. We used the names of the different IMIDs as search terms. The inclusion criteria were as follows: content related to IMIDs, English or Spanish language, and user population consisting of patients and health care consumers, including family and caregivers. The variables analyzed were as follows: app name, type of IMID, platform (Android or iOS), country of origin, language, category of the app, cost, date of the last update, size, downloads, author affiliation, and functionalities.

**Results:**

We identified 713 apps in the initial search, and 243 apps met the criteria and were analyzed. Of these, 37% (n=90) were on Android, 27.2% (n=66) on iOS, and 35.8% (n=87) on both platforms. The most frequent categories were health and well-being/fitness apps (n=188, 48.5%) and medicine (n=82, 37.9%). A total of 211 (82.3%) apps were free. The mean time between the date of the analysis and the date of the most recent update was 18.5 (SD 19.3) months. Health care professionals were involved in the development of 100 (41.1%) apps. We found differences between Android and iOS in the mean time since the last update (16.2, SD 14.7 months vs 30.3, SD 25.7 months) and free apps (85.6% vs 75.8%; respectively). The functionalities were as follows: general information about lifestyles, nutrition, or exercises (n=135, 55.6%); specific information about the disease or treatment (n=102, 42%); recording of symptoms or adverse events (n=51, 21%); agenda/calendar (n=44, 18.1%); reminder medication (n=41, 16.9%); and recording of patient-reported outcomes (n=41, 16.9%). A total of 147 (60.5%) apps had more than one functionality.

**Conclusions:**

IMID-related apps are heterogeneous in terms of functionality and reliability. Apps may be a useful complement to IMID care, especially inpatient education (their most frequent functionality). However, more than half of the IMID apps had not been developed by health care professionals or updated in the last year.

## Introduction

Immune-mediated inflammatory diseases (IMIDs) are systemic conditions characterized by altered immune regulation causing chronic inflammation in specific organs or systems [1]. IMIDs include rheumatologic diseases (eg, rheumatoid arthritis, ankylosing spondylitis, and psoriatic arthritis), digestive diseases (eg, Crohn disease and ulcerative colitis), and dermatologic diseases (eg, psoriasis). It is estimated that 5% to 7% of the world’s population has an IMID [1]. Besides, IMIDs are associated with a high social and health impact in terms of morbidity and mortality, quality of life, and psychological and occupational aspects.

Management and treatment of IMIDs have changed substantially in the last decade, mainly due to the emergence of biological therapies [2]. However, since these new treatments are associated with problems related to administration, toxicity, and adherence, patients should receive adequate training and information. Besides, patients are becoming increasingly active and require more information. A survey conducted in the 27 countries of the European Union revealed that, in the previous 3 months to the survey, 53% of citizens sought online health information related to injury, disease, nutrition, improving health, and similar data [3].

Mobile health (mHealth) technologies incorporating strategies for remote self-management may offer an effective alternative to classic outpatient-based approaches [4]. In particular, broad accessibility to mobile apps enables them to complement health care 24 hours a day, 7 days a week at low cost [5-7]. Apps help to better understand and manage chronic diseases such as IMIDs [8]. Besides, patients with IMIDs are younger than patients with other chronic diseases, and they are more familiar with these technologies [1]. Thus, apps represent an opportunity to improve self-management of care and improve their quality of life.

Health care professionals could play an essential role not only in the review or verification of the contents of these apps but also in their prescription to the best candidates and the recommendation of the most reliable options. However, the lack of studies providing detailed IMID-related app characteristics limits health care professionals’ knowledge in this field. Available IMID apps vary substantially in terms of features, functionality, and reliability [4,9-14]. Up-to-date information about the current situation of apps for patients with IMIDs can be a substantial help to health care professionals in guiding patients and identifying possible risks derived from their use, as well as identifying the needs and directions for future development of these tools. Thus, our objectives were to provide a review of the apps available in the marketplace for patients with IMIDs or caregivers and to identify and describe the main characteristics and functionalities of these apps.

## Methods

We performed an observational, cross-sectional, descriptive study of all the smartphone apps designed for patients with IMIDs available on the iOS and Android platforms.

The methodology used for the selection of the apps followed the PRISMA (Preferred Reporting Items for Systematic Reviews and Meta-Analyses) system. We searched the iOS Apple Store and Android Google Play Store from April 5 to 14, 2021 using the following terms: “ankylosing spondylitis,” “Crohn's disease,” “IBD,” “inflammatory bowel disease,” “immune-mediated inflammatory diseases,” “immune-mediated inflammatory disorders,” “psoriasis,” “psoriatic arthritis,” “rheumatoid arthritis,” and “ulcerative colitis.” As the resultant app list was potentially endless (similar to a Google search), we used the approach followed in previous reviews and performed the screening process until 20 consecutive apps yielded no new potentially relevant apps [15,16].

Two researchers with experience in the analysis of apps, design, and development, and in the management of patients with IMIDs (authors RRJ and VEV) performed identical searches independently. The inclusion criteria were as follows: content related to IMIDs, English or Spanish language, and user population consisting of patients and health consumers, including family or caregivers. Those apps that did not target patients (ie, apps targeting health professionals) were excluded.

The variables analyzed were the name of the app, type of IMID (ankylosing spondylitis, Crohn disease, psoriasis, psoriatic arthritis, rheumatoid arthritis, or ulcerative colitis), platform (Android or iOS), country of origin, language, category of the app, cost, date of the last update, size, downloads, author affiliation, and functionalities. The author’s affiliation was classified as follows: university, health administration or government, hospital/clinic, foundation, patient association, pharmaceutical company, and technology company. Concerning technology companies, we identified two categories depending on whether they were health related or not. Functionalities were further classified into the following categories: information (disease or treatment and lifestyle, nutrition, and exercises), self-diagnosis, disease activity monitoring, symptom or patient-reported outcome (PRO) recording, medication reminders, adherence monitoring, agenda/calendar (a diary or calendar with or without function to record an appointment), contact with health care professionals, and social network. The app search method was developed by the authors and has not been validated. This methodology to analyze app characteristics has been used in other similar studies [4,16,17].

The data were collected from the description and the screenshots available at the app store websites. In cases where one of the variables was not available or there was any doubt, the app was downloaded onto an iPhone 11 (version 14.4.2) or a Xiaomi Redmi Note 7 (version 9.0). For the categorization of author affiliation, we conducted Google searches using the name or the website provided by the app store.

The researchers (RRJ and VEV) evaluated each app independently. Data were analyzed using SPSS Statistics for Windows, Version 21.0 (IBM Corp; descriptive statistics). The homogeneity of the groups was analyzed using a univariate analysis by applying the chi-square test to compare qualitative variables and the *t* test or Mann-Whitney test to compare quantitative variables. A *P* value <.05 was considered statistically significant. Cohen kappa (κ) test was performed to determine the reliability of the data analyzed by these two independent researchers (RRJ and VEV).

The two researchers jointly analyzed those apps again in which the information collected varied between them. Discrepancies were discussed and an agreement was reached.

## Results

### General Characteristics

**Figure 1:**
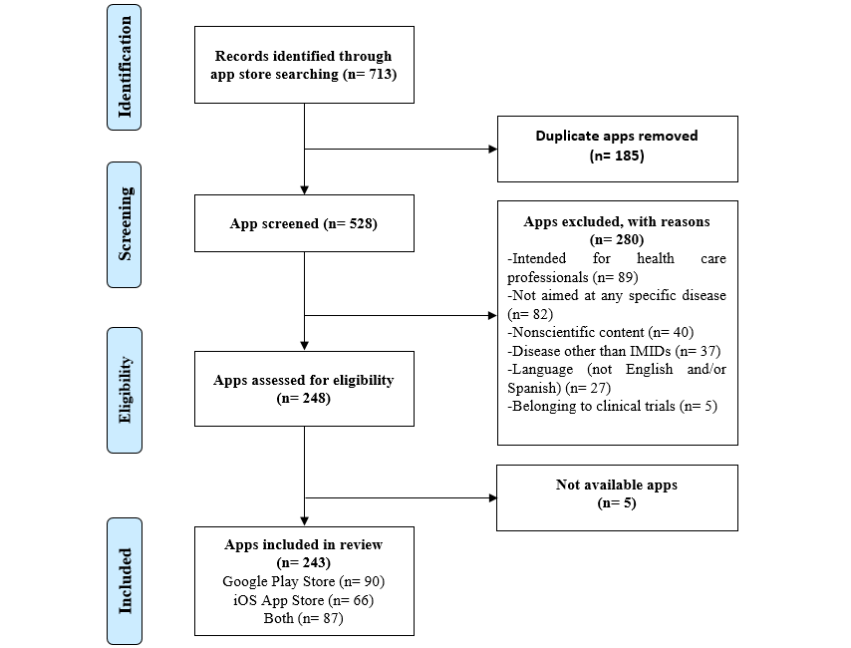
PRISMA (Preferred Reporting Items for Systematic Reviews and Meta-Analyses) flow diagram and app selection. IMID: immune-mediated inflammatory disease.

We identified 713 apps in the initial search in the two app stores; of these, 470 were excluded (Figure 1). We finally analyzed 243 apps: 37% (n=90) on Android, 27.2% (n=66) on iOS, and 35.8% (n=87) on both platforms. Concerning app characteristics, the agreement between the two researchers was excellent (κ=0.868).

Most of the apps belonged to the health and well-being/fitness and medicine categories. The mean time since the last update was 18.5 (SD 19.3) months, and the mean app size was 28.6 (SD 46.9) Mb. A total of 43 (17.7%) apps required payment for use, with a mean price of US $10.30 (SD US $12.00). Concerning the language, 86.8% (n=211) of the apps analyzed were in only one language, and 15 (6.2%) were in three or more languages. A total of 234 (96%) apps were in English, and 11.4% (29/243) were in Spanish. Table 1 shows the remaining general app characteristics.

We found statistically significant differences (*P<.*001) between the Android and iOS apps, as follows: time since the last update, 16.2 (SD 14.7) months vs 30.3 (SD 25.7) months; apps with cost, 14.4% vs. 24.2%; and size, 11.2 (SD 13.9) Mb vs 50.2 (SD 77.9) Mb.

**Table 1:** App characteristics.

Characteristics		Apps (N=243), n (%)
**Store category**		
	Health and well-being/fitness	118 (48.5)
	Medicine	92 (37.9)
	Food and drink	8 (3.3)
	Books and references	7 (3.0)
	Lifestyle	6 (2.5)
	Social networking	4 (1.6)
	Education	3 (1.2)
	Other	5 (2.0)
**Time since last update (months)**		
	<1	23 (9.5)
	2-3	27 (11.1)
	4-6	24 (9.9)
	6-12	37 (15.2)
	12-24	55 (22.6)
	>24	61 (25.1)
	Not available	16 (6.6)
**Downloads**		
	0-100	25 (10.3)
	100-500	32 (13.2)
	500-1000	26 (10.7)
	1000-5000	37 (15.2)
	5000-10,000	19 (7.8)
	10,000-50,000	23 (9.5)
	50,000-100,000	2 (0.8)
	100,000-500,000	10 (4.1)
	>500,000	3 (1.2)
	Not available	66 (27.2)
**Cost**		
	No	200 (82.3)
	Yes	43 (17.7)

### App Author Affiliation

The most frequent type of author affiliation was a nonhealth technology company (n=77, 31.7%; Table 2). The author affiliation was not available in 27.2% (n=66) of apps. Concerning the country of origin, 38.3% (n=93) of apps were from the United States, 8.2% (n=20) were from India, and 7.8% (n=19) were from the United Kingdom.

**Table 2:** App author affiliation.

Characteristics		Apps (N=243), n (%)
**Type of author/developer**		
	Nonhealth technology company	77 (31.7)
	Health-related technology company	57 (23.5)
	Pharmaceutical company	12 (4.9)
	Hospital/clinic	6 (2.5)
	Foundation	18 (7.4)
	Patient association	4 (1.6)
	Health administration/government	2 (0.8)
	University	1 (0.4)
	Not available	66 (27.2)
**Continent of origin**		
	North America	106 (43.6)
	Europe	55 (22.6)
	Asia	32 (13.2)
	Oceania	5 (2.1)
	South America	1 (0.4)
	Not available	44 (18.1)

### Type of IMIDs Targeted by the Apps

A total of 135 (55.5%) apps were specifically aimed at one IMID, and 21 (8.6%) were aimed at all IMIDs (Table 3).

Concerning author affiliation, we found statistically significant differences between specific ankylosing spondylitis–related apps and specific psoriasis-related apps. These differences were found in the number of apps developed by a health-related technology company (50% in psoriasis apps and 6.7% in ankylosing spondylitis), nonhealth technology company (20% in psoriasis apps and 40% in ankylosing spondylitis), and in those apps where this information was not available (30% in psoriasis apps and 53% in ankylosing spondylitis; *P<.*001). No statistically significant differences were found between the rest of the recorded app characteristics and the type of IMIDs.

**Table 3:** Type of immune-mediated inflammatory diseases targeted by the apps.

Characteristics	Apps (N=243), n (%)
Ankylosing spondylitis	56 (23.0)
Crohn disease	93 (38.3)
Psoriatic arthritis	39 (16.0)
Psoriasis	50 (20.6)
Rheumatoid arthritis	111 (45.7)
Ulcerative colitis	89 (36.6)

### App Functionalities

Analysis of the functionalities of the 243 apps revealed that 60.5% (n=147) had more than one functionality. A total of 135 apps offered information about lifestyle, nutrition, and physical exercise, and 102 apps offered information about the disease or its treatment (Table 4). We found that only 6 apps offered information specifically about biological therapies. Table 5 shows the main app functionalities related to the different IMIDs.

The mean number of functionalities per app was 2.1 (range 1-7). No statistically significant differences were found between the number of functionalities and the remaining variables, except for author affiliation. Apps developed by health-related technology companies presented a higher average number of functionalities.

**Table 4:** Functionalities of the apps.

Functionality	App (N=243), n (%)
General information about lifestyle, nutrition, exercise	135 (55.6)
Specific information about the disease or its treatment	102 (42.0)
Recording of symptoms or adverse events	51 (21.0)
Agenda/calendar	44 (18.1)
Medication reminder^a^	41 (16.9)
Recording of patient-reported outcomes	41 (16.9)
Monitoring the activity/severity of the disease	34 (14.7)
Contact with health care professionals	24 (9.9)
Social network	16 (6.6)
Other	36 (14.8)

^a^27 of 41 made it possible to monitor adherence.

**Table 5:** Main app functionalities concerning the different immune-mediated inflammatory diseases.

	Information, n (%)	Recording of symptoms or adverse events, n (%)	Agenda/calendar, n (%)	Reminder medication, n (%)	Recording of PROs^a^, n (%)	Monitoring the activity/severity of the disease, n (%)	Contact with health professionals, n (%)	Social network, n (%)
AS^b^ (n=56)	46 (82.1)	8 (14.3)	8 (14.3)	7 (12.5)	7 (12.5)	4 (7.1)	3 (5.4)	1 (1.8)
CD^c^ (n=93)	68 (73.1)	21 (22.6)	24 (25.8)	19 (20.4)	19 (20.4)	15 (16.1)	9 (9.7)	5 (5.4)
PA^d^ (n=39)	29 (74.5)	8 (20.5)	6 (15.4)	9 (23.1)	7 (17.9)	5 (12.8)	2 (5.1)	1 (2.6)
PS^e^ (n=50)	40 (80.0)	14 (28.0)	8 (16.0)	8 (16.0)	11 (22.0)	4 (8.0)	9 (18.0)	3 (6.0)
RA^f^ (n=111)	80 (72.1)	23 (20.7)	17 (15.3)	20 (18.0)	20 (18.0)	11 (9.9)	7 (6.3)	5 (4.5)
UC^g^ (n=89)	64 (71.9)	22 (24.7)	24 (27.0)	20 (22.5)	16 (18.0)	14 (15.7)	10 (11.2)	7 (7.9)

^a^PRO: patient-reported outcome.

^b^AS: ankylosing spondylitis.

^c^CD: Chron disease.

^d^PA: psoriatic arthritis.

^e^PS: psoriasis

^f^RA: rheumatoid arthritis.

^g^UC: ulcerative colitis.

## Discussion

### Principal Findings

We provide a comprehensive and unique review of IMID-related apps available for patients in the Play Store and Apple Store. The marketplace offers hundreds of solutions, thus making it difficult for the user to filter [17]. Health care professionals are unaware of the real situation of these apps and how they can help patients manage their disease. To the best of our knowledge, this is the only review published so far on the features and functionalities of apps for all IMIDs. The main findings show that functionalities and reliability were heterogeneous. Although most apps offer information, information on biological therapies is scarce. Few apps allow patient interaction, such as recording adverse events, setting alarms for medication, or recording PROs.

We analyzed 243 apps for the most important IMIDs and identified features that may contribute to our understanding of the current landscape and the development of future solutions. Most of the apps targeted rheumatoid arthritis and inflammatory bowel diseases (IBDs). This distribution is proportional to the prevalence of the IMIDs in real life [1]. Among the characteristics of the apps, we highlight the involvement of health care professionals in the development of 41.2% (n=100) of apps. However, 54.3% (n=132) of apps had not been updated in the last year, and the most frequent functionality was that of providing information.

The use of IMID-related apps is still in its infancy [11,18]. The medical fields with the most experience in apps are diabetes, mental health, and cardiovascular disease [7,17,19]. McKay et al [20] carried out a systematic review of apps related to one or more health conditions and found no IMID-related apps. In 2018, there were only 56 IBD-related apps available [21]. In a cohort of 193 German patients with rheumatologic diseases, 91.2% regularly used a smartphone, and 68.4% believed that using medical apps could be beneficial for their health. However, only 4.1% of these patients used health-related apps, of which none were rheumatology-specific [8]. In a similar study performed on 575 French patients diagnosed with rheumatoid arthritis, only 4.7% used a health app [22].

In addition to the limited number of IMID apps, some of the negative characteristics such as a lack of robust scientific evidence prevent clinicians from using health-related apps or recommending them to patients [11,13,23]. It is estimated that only 38% of international consensus statements are completely covered by IBD-related apps focusing on patient education [4]. In a systematic review of self-management apps for persons with arthritic pain, Bhattarai et al [16] observed that a small number of arthritic pain apps offer a comprehensive self-management approach incorporating evidence-based strategies through the Stanford Arthritis Self-Management Program [16].

Currently, there is no specific legislation on the use of the apps and standards or official guidelines on app development. Similarly, there are no official repositories, mainly due to the fast-moving pace of mHealth [5,24]. The Food and Drug Administration launched the Digital Health Precertification Program (Pre-Cert Program) to evaluate mobile-based interventions in real-world conditions [7,25]. Despite the availability of validated tools for assessing the quality of apps, such as the Mobile App Rating Scale [26], there is minimal agreement on methods and the most appropriate criteria for this task [19]. Besides, few IMID-related apps have been developed or validated using these methodologies [14,27,28]. Author affiliation is an important characteristic when searching for a trustworthy app and has been a quality criterion in numerous studies [19]. However, most of the apps did not describe the credentials of the author or professionals who participated in the development. The fact that neither store requires qualifications or affiliations to be listed is one of the main reliability problems of health apps [10,17,29,30]. Thus, external sources, such as the developer’s website if available, are needed. Even so, in 27.2% (n=66) of the apps we reviewed, we could not find any information about the author. We observed that more than half of the author affiliations were related to technology companies, although only 41% (n=100) were identified as involving some type of health professional. Only 7 of the 20 rheumatoid arthritis apps assessed by Luo et al [10] involved a health professional in content development. In another review of apps for medication management, 72.9% of apps were developed by technology companies, 2.1% by academic institutions, and 5.2% by other bodies (governments and nonprofit organizations), and there was insufficient information available about author affiliation in 17.1% [17]. Only 14.6% of apps were developed with the involvement of a health care professional. In a review of apps for rheumatic diseases, authors found that health care professionals were involved in the development of 40% of the apps [12]. Con and Cruz [4] reviewed 26 IBD-related apps and found that health professionals were involved in the development of 19.2% [4].

The level of the update is also a potential cause for concern. Given the rapid advance in IMID care, information must be constantly updated. In our study, we found that more than half of the apps had not been updated in the previous year, while in 25.1% (n=61), the last update had been over 2 years previously. In a review of 20 rheumatoid arthritis apps, only 12 had been updated in the previous year [10]. Concerning other diseases, Collado-Borrell et al [31] observed that 52.4% of cancer-related apps had updated their software within the previous year. We observed that the apps available in the Play Store were more recently updated than those in the App Store. These results contrast with those of studies that have shown the average quality of health apps to be better on iOS than on Android [32].

On the other hand, one of the advantages of these tools is that most health-related apps are free [4,17,31,33], thus favoring their accessibility. Consistent with other studies, we observed that 82.3% (n=200) of IMID-related apps were free [10,17,31,33]. However, in IMID apps requiring payment, the cost is slightly higher (US $10.30) than the average observed in other reviews (US $1.01-4.74) [4,9,10,17,31,33]. Concerning the country of development, the most frequent was the United States, followed by India. Other app review studies have shown a similar distribution [33], likely due to the potential of information and communication technology companies in these countries.

Many of the health-related apps emerged as tools for consulting information, and this is still the most frequent functionality, although 60.5% (n=147) of the apps analyzed had more than one functionality. Our results are consistent with those of other studies and highlight that most apps offer passive functionalities such as information about the disease, treatments, lifestyle, nutrition, and physical exercise. Medication information was the feature most desired by a cohort of 193 patients with rheumatic disease [8]. Mollard et al [11] observed that 25% of apps available for rheumatoid arthritis were exclusively for patient education. Con and Cruz [4] observed that 50% of IBD apps had exclusively diary functionalities that made it possible to track several activities or records, although there was considerable heterogeneity regarding the detail with which symptoms were able to be recorded. In that study, 34.6% of apps only provided information, 11.5% recorded PROs, and 7.7% had two reminder systems. Only 1 app allowed communication between patients and professionals. Similar results were obtained in a review of rheumatoid arthritis apps, in which 50% only allowed symptom tracking and 25% had only provided information [10]. Tabi et al [17] analyzed 328 apps for medication management and observed that 41.2% could share data and reports, although no app allowed bidirectional communication with health professionals.

Several studies have shown the usefulness of apps for improving adherence [17,34]. This feature is even more important in drugs that are not administered daily, such as biological therapies, as delays in administration may be more frequent. For instance, 20% to 40% of patients with IBD are nonadherent. Alerts or reminders to take medication are the most frequent feature of apps and improve adherence. In general, apps are the preferred method for reminding patients to take their medication regularly [8]. However, in our review, only 16.9% (n=41) of apps have this functionality, and only 11.1% (n=27) enable medication adherence to be monitored.

Encouraging enthusiastic patients to engage with apps regularly is another barrier [11]. For example, many patients download an app for the sole purpose of obtaining health information. However, after initial use of the app, there is unlikely to be continual engagement once the desired information is obtained [11]. Long-term engagement can be problematic, with app interventions progressively becoming less effective over time [7]. Some functionalities involving patient participation, such as sending messages and gamification, can also improve long-term engagement [19]. Gamification is a specific characteristic of mHealth interventions that could favor behavioral change and increase user engagement. However, we only found 1 app with the functionality of gamification in our review.

### Limitations and Strengths

A possible limitation is that we did not assess the privacy and confidentiality of apps since this information is not available in most cases. The relationship between the use of health apps and privacy risk is understudied [10]. Most reviews and studies that assess app quality do not analyze this aspect [19,20]. In fact, the number of health apps that define privacy policies is low [35]. Nevertheless, to our knowledge, this is the first review to cover most IMIDs and analyze their characteristics in detail. We believe that our review can provide health professionals with a real-world picture of the apps available for patients with IMIDs and help them to identify strengths and weaknesses. We have observed that there are a large number of free apps in English available for patients diagnosed with IMIDs, especially for consulting information. However, when recommending apps or instructing patients to search for them, health care professionals should be aware of some critical factors. Professionals and patients should check the author’s affiliation and the update date, as there are many IMID apps with nonproven developers and without updates. Future legislation for apps should focus specifically on bridging these gaps.

### Conclusions

This review provides health professionals with an overview of available IMID-related apps and their characteristics. IMID-related apps continue to be heterogeneous in terms of their functionalities and reliability. Apps may act as a useful complement to IMID care, especially in patient education, as this is their most frequent functionality. However, more than half of the IMID apps had not been developed by health care professionals or updated in the previous year.

## Abbreviations

IBD: inflammatory bowel disease
IMID: immune-mediated inflammatory disease
mHealth: mobile health
PRISMA: Preferred Reporting Items for Systematic Reviews and Meta-Analyses
PRO: patient-reported outcome
